# Development and validation of a model for temporal lobe necrosis for nasopharyngeal carcinoma patients with intensity modulated radiation therapy

**DOI:** 10.1186/s13014-019-1250-z

**Published:** 2019-03-12

**Authors:** Jiazhou Wang, Yibing Miao, Xiaomin Ou, Xiaoshen Wang, Xiayun He, Chunying Shen, Hongmei Ying, Weigang Hu, Chaosu Hu

**Affiliations:** 1 Department of radiation oncology, Fudan University Shanghai Cancer Center, Shanghai, China; 2 Department of Oncology, Shanghai Medical College, Fudan University, Shanghai, China

**Keywords:** Temporal lobe necrosis, Nasopharyngeal carcinoma, Normal tissue complication probability

## Abstract

**Purpose:**

To develop and validate a quantitative complication model of temporal lobe necrosis (TLN). To analyze the effect of clinical and dosimetric factors on TLN.

**Patients and methods:**

In this study the prediction model was developed in a training cohort that consisted of 256 nasopharyngeal carcinoma (NPC) patients from January 2009 to December 2009. Dosimetric and clinical factors were extracted for model building. Dosimetric factors including the maximum dose, minimum dose, mean dose, dose covering specific volume and dose of percentage volume. Clinical factors include age, gender, T/N-stage, overall stage, diabetes and hypertension. LASSO (least absolute shrinkage and selection operator) regression model was used for feature selection, and prediction model building. A testing cohort containing 493 consecutive patients from January 2010 to December 2010 was used for model validation. The performance of the prediction model was assessed with respect to its calibration, discrimination.

**Results:**

The prediction model, which consisted of two dosimetric features (D0.5cc and D10), is significantly associated with LN status (*P* < .001 for both training and testing cohorts). None of clinical factors show direct prediction value. The model shows good discrimination, with a C-index of 0.685 (95% CI: 0.6048–0.765) on testing set, and a consistent trend in calibration on testing set.

**Conclusion:**

This study presents a prediction model can be conveniently used to facilitate the individualized prediction of TLN in patients with NPC. Clinical factors have no direct impact on TLN.

**Electronic supplementary material:**

The online version of this article (10.1186/s13014-019-1250-z) contains supplementary material, which is available to authorized users.

## Introduction

In patients with nasopharyngeal carcinoma (NPC), an impressive local control rate has been achieved, and as a result, the late toxicities of long-term survival after radiation therapy have become an important concern. Temporal lobe necrosis (TLN) is one major complication observed in NPC patients after radiation therapy. Because of the nearness in the anatomical structure of the TL and nasopharynx, TLs are prone to receiving high doses of radiation, causing cerebral tissue damage. Typical symptoms of TLN include dizziness, lethargy, debilitation, personality change, pressure symptoms, and epileptic attacks. Further failure of cognition can irreversibly impair patients’ quality of life.

Intensity-modulated radiation therapy (IMRT) improves the physical dose distribution compared to two-dimensional RT (2DRT), and with this dose improvement, the corresponding incidence of TLN decreases significantly [1, 2]. Indeed, the incidence of TLN after IMRT from different centers varies from 3 to 14% [3–8]. QUANTEC suggested that the dose limit for TL D_max_ ≤ 60 Gy and V_65Gy_ ≤ 1% are commonly accepted in clinical use. However, the QUANTEC limits are based on past experience from the 3DCRT or 2DRT era and may not be suitable for IMRT. A retrospective analysis performed by Su et al. [9]. revealed that IMRT D_max_ < 68 Gy or D_1cc_ < 58 Gy for the TL is relatively safe. Their study covered a series of dosimetric factors. However, following study of Su et al., pointed out rV40 and aV40 (relative and absolute volume receiving dose higher than 40 Gy) could better predict TLN [10]. Sun et al. suggested a dose limitation of D_0.5cc_ < 69 Gy based on an analysis of 506 patients [11]. A case-controlled study performed by Zhou found that both focal high dose and moderate dose to large volume should be considered [12].

Most previous studies focused on the dose only, and clinical factors have been seldom discussed. In clinical practice, the overall condition of the patient should be taken into consideration. Although there have been several previous publications looking at dosimetric and patient factors in determining a safe dose to the temporal lobes during NPC IMRT treatment. There was no a comprehensive and quantitative analysis.

The purpose of this study was to generate a model to predict TLN occurrence using clinical and dosimetric factors. And to access model performance on a testing data set.

## Methods

**Fig. 1:**
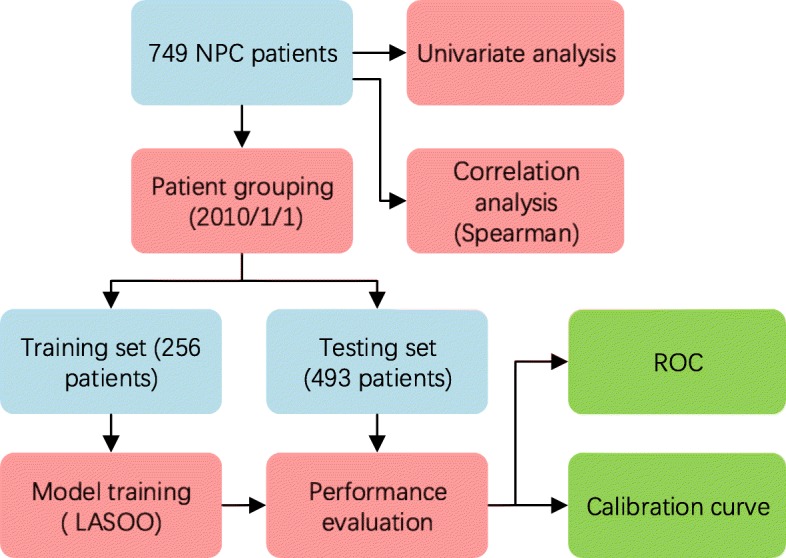
The workflow of this study

The workflow of this study is presented in Fig. 1. Patients were divided into training set and testing set base on patients’ treatment time. A LASSO modeling method was performed to build a prediction, and the model performance was evaluated by ROC and calibration curve.

### Patients

From January 2009 to December 2010, 749 NPC patients receiving IMRT in our center were included in the study. All the patients completed full course radiotherapy and were metastatic free. Patients were followed up after radiotherapy every 3 months for the first 2 years, every 6 months from year 2 to year 5, and annually thereafter. The last follow-up date was July 2015. After a median follow-up of 48.8 months (range 3.5–75.1 months), 38 out of 749 (5.07%) patients were diagnosed with TLN based on magnetic resonance imaging (MRI). Twenty-six of the patients had a unilateral lesion, and 12 had bilateral lesions. TLN grading was performed according to RTOG/EORTC late radiation morbidity scoring schema. Most of the TLN cases (32 out of 38) were of grade 1 (mild symptoms). Two cases were grade 2, and four cases were grade 3.

To extract the precise dose parameters, re-delineation of the TLs was performed on all cases in this study. Contouring of the TL referred to the suggestion by Sun et al. [13] and included the hippocampus, parahippocampal gyrus and uncus. DVH curves of the TLs were exported from the original treatment plans on the Pinnacle (Pinnacle 3; Philips Corp, Fitchburg, WI) treatment planning system (TPS). Dose parameters including the maximum dose (D_max_), minimum dose (D_min_), mean dose (D_mean_), dose covering specific volume (D_vs_), and dose of percentage volume (D_vp_) were derived from the exported DVH curves. Clinical factors included age, gender, T/N-stage, overall stage, diabetes and hypertension. Clinical characteristics were retrospectively reviewed from a clinical database under the approval of the Institutional Review Board. All patients were staged according to the 7th American Joint Committee on Cancer/Union for International Cancer Control staging system.

### Treatment protocol

Planning target delineation follows the definition of ICRU 50 and 62 reports. The gross tumor volume (GTV) covers primary tumor and metastatic lymph nodes. The clinical target volume (CTV) covers the entire nasopharynx, parapharyngeal space, clivus, the base of skull, pterygoid fossa, the posterior half of ethmoidal sinus, inferior sphenoid sinus, and the posterior edge of nasal cavity and maxillary sinuses. The planning target volume (PTV) extends 3–5 mm around the GTV or CTV. A total dose of 66 Gy in 30 fractions is delivered to T1 and T2 and 70.4 Gy in 32 fractions to T3 and T4 via the simultaneous integrated boost-IMRT (SIB-IMRT) technique. Sixty-six Gy in 30–32 fractions is delivered to the metastatic lymph nodes. For high-risk and low-risk CTV, total doses of 60 and 54 Gy are delivered in 30–32 fractions, respectively.

Stage I-IIA patients were treated with radiation alone. Stage IIB-IVB received the recommended cisplatin-based chemotherapy. In addition, 634 of 749 (84.6%) underwent chemotherapy. Of these, 555 patients (74.1%) underwent induction chemotherapy: 247 (33.0%) patients received concurrent chemotherapy, and 209 (27.9%) patients received adjuvant chemotherapy.

### Statistics and modeling

All statistics analysis was based on temporal lobe, so the sample number were double. A univariate analysis was performed to analyze the impact of each features. For continuous variables, a logistic regression was used. For binary variables, a chi-square test was used. A spearman correlation coefficient for all dosimetric and clinical factors were calculated to analyze the relation between factors.

The patient data were divided by date (2010-01-01). The prior portion comprised the training set (256), and the latter portion composed the testing set (493). The two lobes were analyzed separately, and the quantities were doubled. The reason to choose this time point is the event number is almost equal in training (40) and testing set (39). The different TLN probability in training and testing set may caused by the difference in the follow-up time (median follow-up: training 55.6 months, testing 38.1 months). We believe using longer time as training set was more appropriate.

A cross-validation LASSO was used in model development. The model prediction power was assessed on testing set by the area under the curve (AUC) and calibration curve. Briefly, training set was randomly spliced into 5 parts (5 folds). Four parts were used to create model and the last part was used to find best lambda value in penalized logistic regression model. After best lambda value was selected, the final model was established in whole training set. More modeling details are described in Additional file 1: Supplement A.

Meanwhile to evaluate the bias came from data splitting. A 3-fold cross-validation was used to separated data into training and testing. And same processing was applied base on this data splitting. The detail and result of this evaluation were described in Additional file 1: Supplement B.

In order to analyze the prediction power of diagnosis, treatment and dose features, four models with same strategy were developed, including diagnosis features only, treatment features only, diagnosis and treatment features, and dose features only. Diagnosis features include age, gender, T/N-stage, overall stage, diabetes and hypertension. Treatment feature includes chemotherapy. Dose features contains all dosimetric factors.

Data preprocessing and analysis were accomplished using R language. The package ‘glmnet’ was used for modeling and validation.

## Results

### Patient characteristics and univariate analysis

The results and patients’ characters are showed in Table 1. Regarding the clinical factors, only T-stage significantly affected (training *p* = .045, testing *p* = .002) TLN occurrence. The median latency for TLN is 39.5 months (range 3.5–74.3 months). The number for different grade of necrosis is 32/2/4/0 for grading 1/2/3/4. All dosimetric factors were significant related to TLN (*p* < .05), except D80 (*p* = .075). All dosimetric factors have relative high interrelationship. Only 14 out 136 (10%) matched pairs have correlation coefficient less than 0.3. T stage is correlation to all dosimetic factors (*ρ* = 0.32~0.47). The detail was provided in Additional file 1: Supplement C.

**Table 1:** Patient characteristics and univariate analysis

Characteristics	Patient number	Training Cohort (512 samples)			Testing Cohort (986 samples)		
	749 (100)	Necrosis (+)	Necrosis (−)	*P*	Necrosis (+)	Necrosis (−)	*P*
Age, mean ± SD, years	48.7 ± 12.0	48.8 ± 9.36	47.4 ± 11.7	.453	50.3 ± 10.6	49.2 ± 12.3	.595
Gender				.528			.131
Male	504 (67.3)	29 (5.6)	369 (72.1)		27 (2.7)	683 (69.3)	
Female	195 (26.0)	11 (2.1)	103 (20.1)		12 (1.2)	264 (26.8)	
Diabetes				.526			.551
Yes	41 (5.5)	3 (0.6)	19 (3.7)		1 (0.1)	59 (6.0)	
No	708 (94.5)	37 (7.2)	453 (88.5)		38 (3.9)	888 (90.1)	
Hypertension				1.000			.131
Yes	79 (10.6)	4 (0.8)	42 (8.2)		1 (0.1)	111 (11.3)	
No	670 (89.5)	36 (7.0)	430 (84.0)		38 (3.9%)	836 (84.8)	
T-stage				.045*			.002*
T1	215 (28.7)	5 (1.0)	119 (23.2)		5 (0.5)	301 (30.5)	
T2	244 (32.6)	16 (3.1)	172 (33.6)		9 (0.9)	291 (29.5)	
T3	194 (25.9)	7 (1.3)	89 (17.4)		19 (1.9)	263 (26.7)	
T4	96 (12.8)	12 (2.3)	82 (16.0)		6 (0.6)	92 (9.3)	
N-stage				.421			.155
N0	109 (14.5)	4 (0.8)	74 (14.4)		4 (0.4)	136 (13.8)	
N1	332 (44.3)	24 (4.7)	192 (37.5)		24 (2.4)	424 (43.0)	
N2	216 (28.8)	10 (2.0)	168 (32.8)		10 (1.0)	244 (24.7)	
N3	92 (12.28)	2 (0.4)	38 (7.4)		1 (0.1)	143 (14.5)	
Overall stage				.913			.763
I	45 (6.01)	1 (0.2)	25 (4.9)		0 (0)	64 (6.5)	
II	226 (30.2)	17 (3.3)	133 (26.0)		8 (0.8)	294 (29.8	
III	295 (39.4)	8 (1.6)	196 (38.3)		24 (2.4)	362 (36.7)	
IVA	91 (12.15)	12 (2.3)	80 (15.6)		6 (0.6)	84 (8.5)	
IVB	92 (12.3)	2 (0.4)	38 (7.4)		1 (0.1)	143 (14.5)	
Induction chemotherapy				.477			.832
Yes	550 (73.4)	30 (5.9)	322 (62.9)		12 (1.2)	264 (26.8)	
No	194 (25.9)	10 (2.0)	150 (29.3)		27 (2.7)	683 (69.3)	
Concurrent chemotherapy				1.000			.080
Yes	334 (44.6)	20 (3.9)	242 (47.3)		35 (3.5)	723 (73.3)	
No	415 (55.4)	20 (3.9)	230 (44.9)		4 (0.4)	224 (22.7)	
Adjuvant chemotherapy				.662			.418
Yes	226 (30.2)	11 (3.1)	109 (21.3)		19 (1.9)	387 (39.2)	
No	523 (69.8)	29 (5.7)	363 (70.9)		20 (2.0)	560 (56.8)	
Predicted probability, median (95% range)		9.8 (5.0–19.1)	6.7 (3.6–13.9)	<.001*	8.4 (5.1–25.3)	6.4 (3.1–15.4)	<.001*

All statistics were based on temporal lobe number. *P* value is derived from the univariable association analyses between each of the clinical variables and TLN status. For binary variables, a chi-square test was used

*P value <.05

### Feature selection and prediction model building

**Fig. 2:**
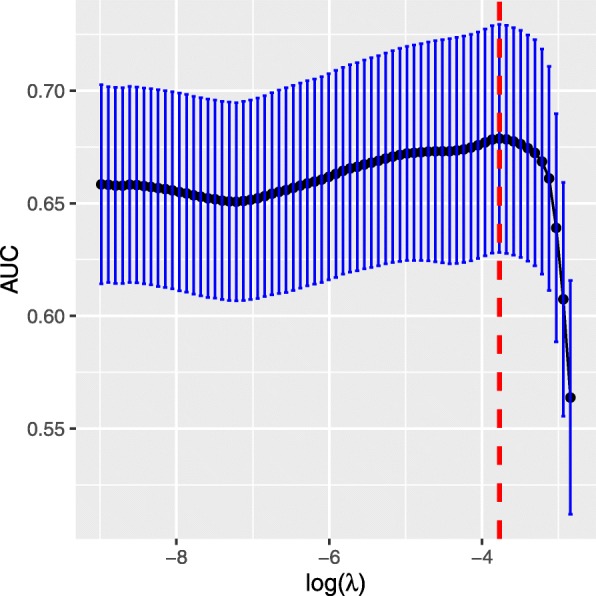
Feature selection using the least absolute shrinkage and selection operator (LASSO) binary logistic regression model. Tuning parameter (λ) selection in LASSO used 10-fold cross-validation via minimum criteria. The area under the receiver operating characteristic (AUC) was plotted versus log(λ). The red dot lines were draw at the optimal values by using minimum criteria. The best AUC is 0.6787 with standard deviation 0.05

Of the features, 27 were reduced to 2 predictors on the basis of 256 patients in the primary cohort (Fig. 2), and they were with nonzero coefficients in the LASSO logistic regression model. The final enrolled features are D0.5cc and D10. These features were presented in the prediction model formula. The unit for D0.5cc and D10 was cGy.

$$ Probability\ of\ Temporal\ Lobe\ Necrosis=\frac{1}{1+\exp \left(7.36-0.00036{D}_{0.5 cc}-0.00054{D}_{10}\right)} $$

### Validation of prediction model

**Fig. 3:**
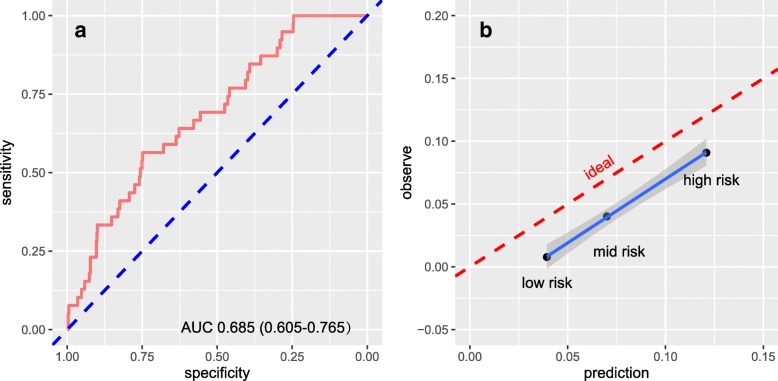
The receiver operating characteristic curve and calibration curves for testing set. **a** The receiver operating characteristic curve with AUC 0.6849 (95% CI: 0.6048–0.765). **b** The calibration curve. ‘Low risk’ is the TLN risk less than 5%; ‘mid risk’ is the TLN risk between 5 and 10%; ‘high risk’ is the TLN risk large than 10%

The receiver operating characteristic curve and calibration curve for testing set was present in Fig. 3. Here the AUC for testing set is 0.6849 (95% CI: 0.6048–0.765). Although there is a deviate between ideal line and calibration curve, which we will discuss it later. The trend of the prediction is consistent with observation. The different dataset splitting has similar results. Detail was provided in Additional file 1: Supplement B.

### Prediction power analysis

The prediction power analysis was presented in Table 2. Without dose features, other features can only provide prediction power less than 0.55. The most powerfull prediction feature is the T stage. Because the T stage is corrlated with dose and prescrption. When T stage and dose are analyzed together, T stage will be removed by LASSO model. More details are described in Additional file 1: Supplement D.

**Table 2:** Prediction power analysis

Model	Training AUC	Testing AUC
Diagnosis	0.55 (0.47–0.63)	0.64 (0.57–0.73)
Treatment	0.46 (0.38–0.54)	0.57 (0.52–0.62)
Diagnosis+Treatment	0.55 (0.47–0.63)	0.64 (0.57–0.73)
Dose	0.68 (0.60–0.76)	0.68 (0.60–0.76)

### Clinical use

**Fig. 4:**
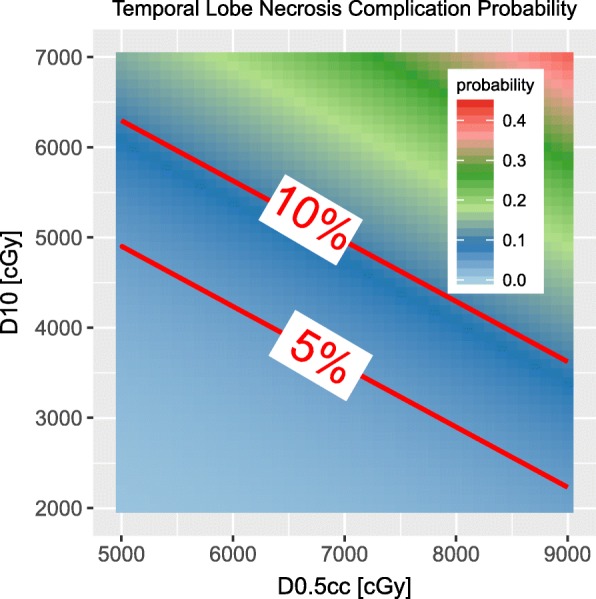
The probability of the temporal lobe necrosis with two dose indices

To facilitate clinical use, a probability map with two dose factors is presented in Fig. 4. Two regions are delineated by our model. Dose in first region will have TLN probability less than 5%. And the 2nd region is 10%.

## Discussion

In this study, both clinical and dosimetric factors were evaluated. A robust modeling method was implemented to get final model. As we know, this study is the first attempt to derive a quantitative complication model for TLN. This is a logical extension of the previous statistical studies.

The results show that only physical dose parameters were reliable factors for the prediction of TLN. Although, the clinical factor T-stage is strongly correlated with TLN in univariate analysis. It was not selected into final prediction model. This may cause by the strong correlation between dose and T-stage (0.42, Spearman’s correlation of T-stage and TL Mean Dose). The dosimetric factor seem as more direct cause. Moreover, when considering advanced T stage (T3, T4) patients alone, the correlation was weaker (0.335) when the prescription dose was identical.

Diabetes is accompanied by a biological change in the microvascular environment. Its enhancement of TLN supports the hypothesis put forward by Belka that vessel damage is a cerebral toxicity inducing factor [14]. Because of the limited number of patients, further investigation is expected to draw a statistically persuasive conclusion. Concurrent chemotherapy exhibited a similar situation and induction or adjuvant chemotherapy showed no positive effect. These results were consistent with those obtained in Lee’s study [15].

Dosimetric parameters are strongly correlated with each other. In variable selection, collinearity causes competition among predictors and makes arbitrary decisions in choice [16]. The prediction variables were selected via LASSO [17]. LASSO is another approach that can be used to select highly correlated variables for which strongly correlated predictors tend to be in and out together [18]. More advanced modeling strategies, such as random forests, are also alternatives and may achieve more powerful predictions. However, considering the accessibility required for clinical purposes, they may not be a first choice.

Previous studies provided different conclusions regarding dosimetric variables. In addition to various specific cutoff limitation points, a focal high dose [9, 11, 17] or relatively large volume dose [10, 12] are also critical differences. The affecting factors could include TL delineation, treatment strategy, and follow-up period. The posterior and superior margins of the TL are not precisely shown on the CT images, resulting in variances in the dose description of the TL. In our study, although lots dosimetric factors were correlated to TLN, the final selected features were D0.5cc and D10. This result have demonstrated that a hot spot and a small region (10% of the TL) have most effects on the TLN.

The low incidence of TLN makes clinical control difficult. After a median follow-up of 48.8 months, some of the current necrosis-free patients will likely show symptoms of TLN in the future. With an extended follow-up period, more persuasive findings may be obtained. This is the main reason that the TLN incidence rate difference in our training and testing set. This also cause a deviation of the calibration curve. Additionally, although necrosis occurs only within a small region, the dose administered to the entire TL is usually analyzed. A compromise approach is to define a ‘sub-TL’ ROI that delineates only the front TL because nearly all cases of TLN occur in the anterior and lower parts of the TL. Applying this approach might generate a clearer TLN nomogram.

## Conclusion

This study presents a prediction model can be conveniently used to facilitate the individualized prediction of TLN in patients with NPC. Clinical factors have no direct impact on TLN.

## Additional file

Additional file 1: **Supplement A.** The detail of the model building. **Supplement B.** Data splitting bias evaluation, method and results. **Supplement C.** Univariate analysis for dosimetric factors and correlation analysis. **Supplement D.** Support material for the prediction power. (DOCX 1997 kb)

## Abbreviations

LASSO: Least absolute shrinkage and selection operator
NPC: Nasopharyngeal carcinoma
TLN: Temporal lobe necrosis

## References

[1] Peng G, Wang T, K-y Y, Zhang S, Zhang T, Li Q, Han J, Wu G (2012). A prospective, randomized study comparing outcomes and toxicities of intensity-modulated radiotherapy vs. conventional two-dimensional radiotherapy for the treatment of nasopharyngeal carcinoma. Radiother Oncol.

[2] Zhou G-Q, Yu X-L, Chen M, Guo R, Lei Y, Sun Y, Mao Y-P, Liu L-Z, Li L, Lin A-H (2013). Radiation-induced temporal lobe injury for nasopharyngeal carcinoma: a comparison of intensity-modulated radiotherapy and conventional two-dimensional radiotherapy. PLoS One.

[3] Bakst RL, Lee N, Pfister DG, Zelefsky MJ, Hunt MA, Kraus DH, Wolden SL (2011). Hypofractionated dose-painting intensity modulated radiation therapy with chemotherapy for nasopharyngeal carcinoma: a prospective trial. Int J Radiat Oncol Biol Phys.

[4] Kwong DL, Sham JS, Leung LH, Cheng AC, Ng W, Kwong PW, Lui W, Yau C, Wu P, Wei W (2006). Preliminary results of radiation dose escalation for locally advanced nasopharyngeal carcinoma. Int J Radiat Oncol Biol Phys.

[5] Kam MK, Teo PM, Chau RM, Cheung K, Choi PH, Kwan W, Leung S, Zee B, Chan AT (2004). Treatment of nasopharyngeal carcinoma with intensity-modulated radiotherapy: the Hong Kong experience. Int J Radiat Oncol Biol Phys.

[6] Xiao WW, Huang SM, Han F, Wu SX, Lu LX, Lin CG, Deng XW, Lu TX, Cui NJ, Zhao C (2011). Local control, survival, and late toxicities of locally advanced nasopharyngeal carcinoma treated by simultaneous modulated accelerated radiotherapy combined with cisplatin concurrent chemotherapy. Cancer.

[7] Bucci M, Xia P, Lee N, Fishbein N, Kramer A, Weinberg V, Akazawa C, Cabrera A, Fu K, Quivey J (2004). Intensity modulated radiation therapy for carcinoma of the nasopharynx: an update of the UCSF experience. Int J Radiat Oncol Biol Phys.

[8] Millican E, Schwarz J, Thorstad W (2009). Retrospective review of temporal lobe necrosis following IMRT for nasopharyngeal carcinoma. Int J Radiat Oncol Biol Phys.

[9] Su S-F, Huang Y, Xiao W-w, Huang S-M, Han F, Xie C-m, Lu T-X (2012). Clinical and dosimetric characteristics of temporal lobe injury following intensity modulated radiotherapy of nasopharyngeal carcinoma. Radiother Oncol.

[10] Su S-F, Huang S-M, Han F, Huang Y, Chen C-Y, Xiao W-W, Sun X-M, Lu T-X (2013). Analysis of dosimetric factors associated with temporal lobe necrosis (TLN) in patients with nasopharyngeal carcinoma (NPC) after intensity modulated radiotherapy. Radiat Oncol.

[11] Sun Y, Zhou G-Q, Qi Z-Y, Zhang L, Huang S-M, Liu L-Z, Li L, Lin A-H, Ma J (2013). Radiation-induced temporal lobe injury after intensity modulated radiotherapy in nasopharyngeal carcinoma patients: a dose-volume-outcome analysis. BMC Cancer.

[12] Zhou X, Ou X, Xu T, Wang X, Shen C, Ding J, Hu C (2014). Effect of dosimetric factors on occurrence and volume of temporal lobe necrosis following intensity modulated radiation therapy for nasopharyngeal carcinoma: a case-control study. Int J Radiat Oncol Biol Phys.

[13] Sun Y, Yu XL, Luo W, Lee AW, Wee JT, Lee N, Zhou GQ, Tang LL, Tao CJ, Guo R (2014). Recommendation for a contouring method and atlas of organs at risk in nasopharyngeal carcinoma patients receiving intensity-modulated radiotherapy. Radiother Oncol.

[14] Belka C, Budach W, Kortmann R, Bamberg M (2001). Radiation induced CNS toxicity–molecular and cellular mechanisms. Br J Cancer.

[15] Lee AW, Ng W, Hung W, Choi C, Tung R, Ling Y, Cheng PT, Yau T, Chang AT, Leung SK (2009). Major late toxicities after conformal radiotherapy for nasopharyngeal carcinoma—patient-and treatment-related risk factors. Int J Radiat Oncol Biol Phys.

[16] Harrell FE. Regression modeling strategies. BIOS. 2014;330:78–9.

[17] Kong C, Zhu XZ, Lee TF, Feng PB, Xu JH, Qian PD, Zhang LF, He X, Huang SF, Zhang YQ (2016). LASSO-based NTCP model for radiation-induced temporal lobe injury developing after intensity-modulated radiotherapy of nasopharyngeal carcinoma. Sci Rep.

[18] Zou H, Hastie T (2005). Regularization and variable selection via the elastic net. J R Stat Soc Ser B (Stat Methodol).

