# Supplementary material for: Development and validation of a model for temporal lobe necrosis for nasopharyngeal carcinoma patients with intensity modulated radiation therapy
Source: Radiat Oncol. 2019 Mar 12;14:42. doi: 10.1186/s13014-019-1250-z (PMC6416868; doi:10.1186/s13014-019-1250-z)
Supplement: Supplementary file 1 — Supplement A. The detail of the model building. Supplement B. Data splitting bias evaluation, method and results. Supplement C. Univariate analysis for dosimetric factors and correlation analysis. Supplement D. Support material for the prediction power. (DOCX 1997 kb) [file 13014_2019_1250_MOESM1_ESM.docx]

**Supplement A. The detail of the model building.**

The modeling process is presented in figure s1. First use 5 fold cross-validation to split train set 256 patients into 205 and 51 patients. A penalized logistic regression model was fitted on these 205 patients. The fitted model was used to predict the probability of the 41 patients. Calculate the performance of this penalty setting. This process was repeated 200 times to find a stable penalty setting (lambda value). Then this lambda value is used to build the final model on the training set (256 patients). The final model performance was evaluated on testing set (493 patients) to reduce the variability in cross validation.

Figure s1. The modeling workflow

**Supplement B. Data splitting bias evaluation, method and results.**

Because the training and testing data splitting was arbitrary. To evaluate the bias caused by this splitting, a 3-fold cross validation was used. Due to low incidence rate of TLN, 3-fold cross validation can keep enough events in testing set. This process put all data together, then randomly splited the cohort into 3 parts. Then same modeling method was applied.

Here is results. The figure s2 showed the lasso features selection. Although the some AUC (cv3) have some difference with figure 2. The AUC curve have large flat region, which means AUC can keep a stable level with feature reducing. And the results of the ROC curve were presented in figure. The AUC value are presented in table s1. The testing set value was close to the value in manuscript (0.685. 95% CI: 0.6048-0.765).


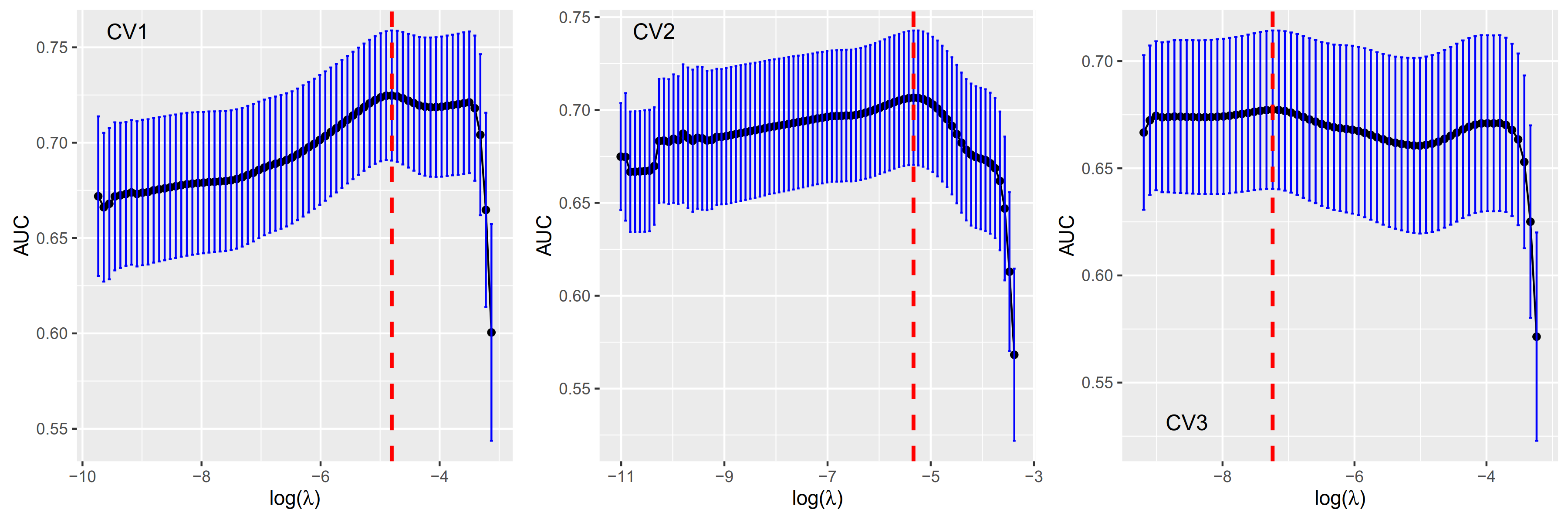


Figure s2. Feature selection using the least absolute shrinkage and selection operator (LASSO) binary logistic regression model. Tuning parameter (λ) selection in LASSO used 5-fold cross-validation via minimum criteria. The red dot lines were draw at the optimal values by using minimum criteria.


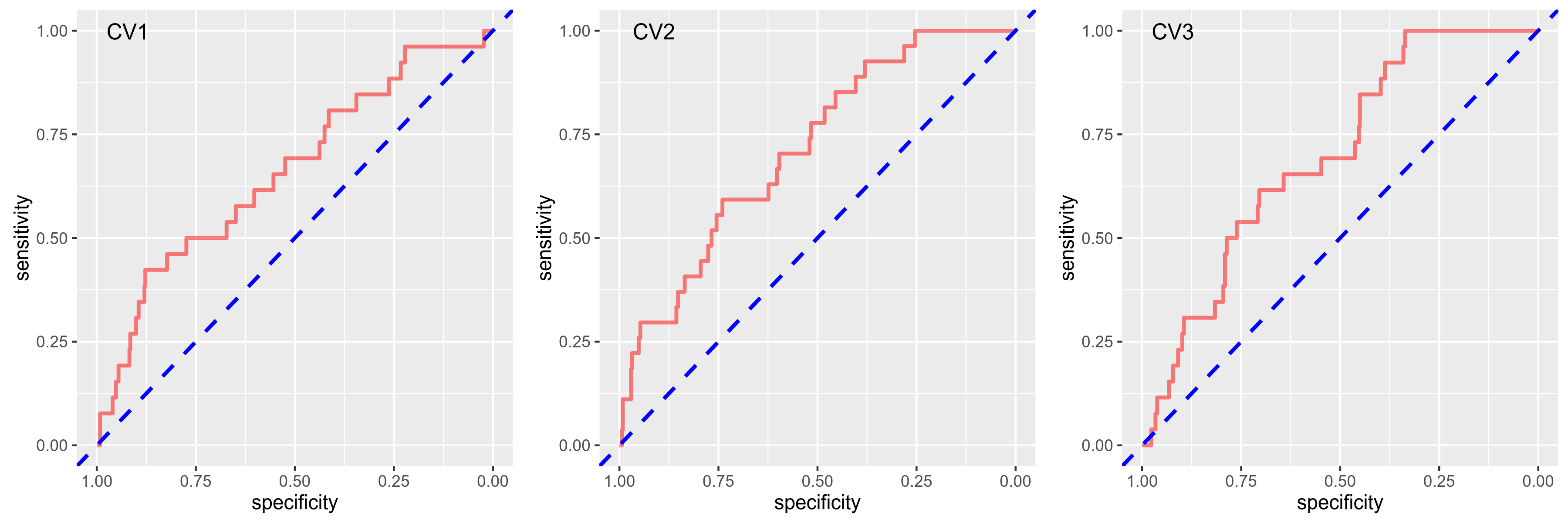


Figure s3. ROC curves for 3-fold cross validation

Table s1. The AUC value for cross validation

|  | Training set | Testing set |
| --- | --- | --- |
| cv1 | 0.725 (0.711, 0.759) | 0.660 (0.547, 0.774) |
| cv2 | 0.707 (0.671 0.743) | 0.714 (0.622, 0.714) |
| cv3 | 0.677 (0.714, 0.640) | 0.694 (0.607, 0.784) |
| Mean | 0.703 | 0.689 |

**Supplement C. Univariate analysis for dosimetric factors and correlation analysis.**

The univariate analysis for dosimetric factors was showed in table s2. The spearman correlation was show in figure s4.

Table s2, The univariate analysis for dosimetric factors.

| Dose factors | p-value |
| --- | --- |
| MeanDose | 6.71E-06 |
| MaxDose | 5.13E-08 |
| D0.5cc | 4.68E-10 |
| D1cc | 5.44E-10 |
| D2cc | 6.66E-10 |
| D3cc | 2.52E-09 |
| D5cc | 9.51E-09 |
| D10cc | 3.04E-07 |
| D5 | 1.03E-09 |
| D10 | 3.44E-09 |
| D20 | 1.08E-06 |
| D30 | 3.30E-05 |
| D40 | 0.000404 |
| D50 | 0.001229 |
| D60 | 0.005522 |
| D70 | 0.018368 |
| D80 | 0.075088 |

.


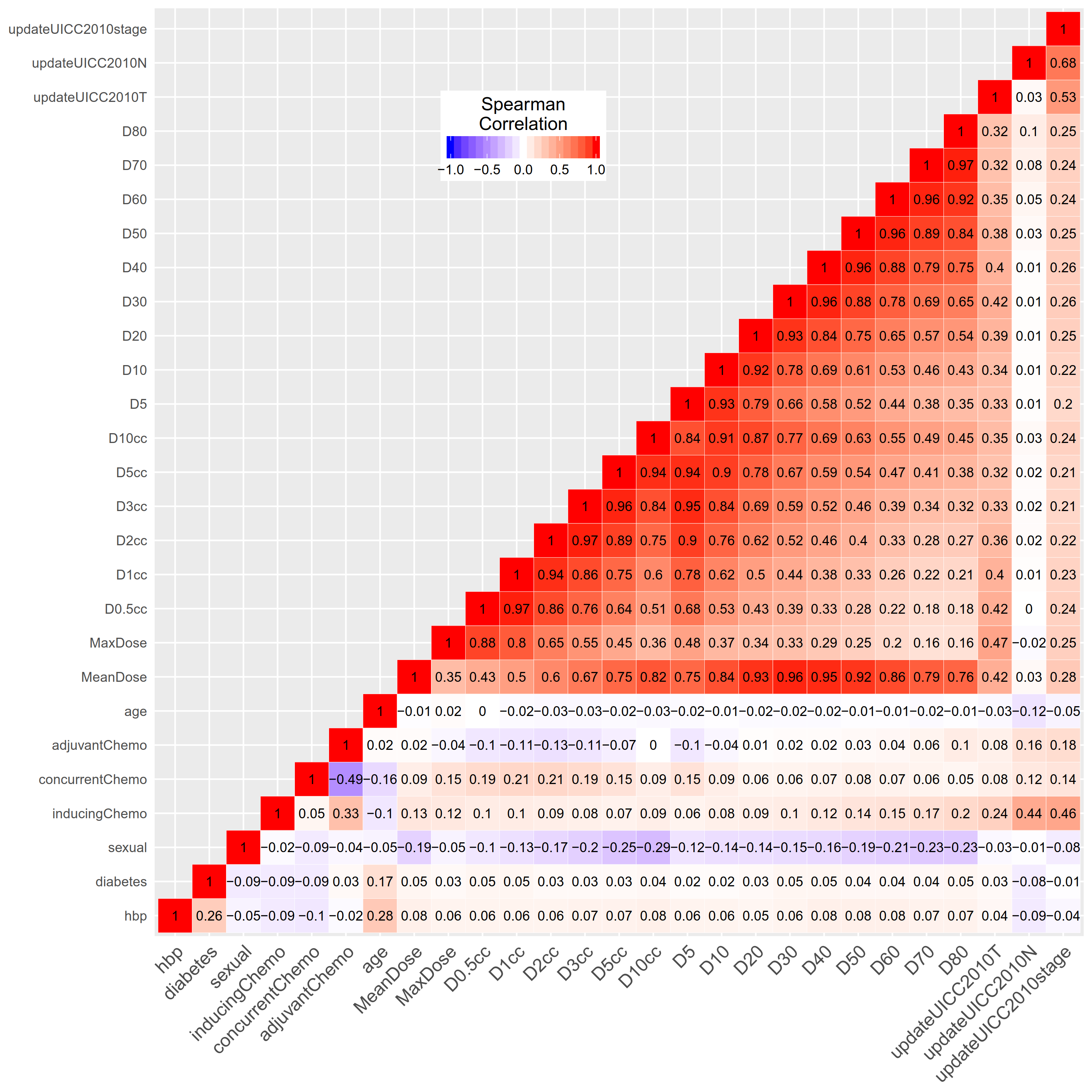


Figure s4. The spearman correlation map for different features.

**Supplement D. Support material for the prediction power.**

Other model performance. Figure s4 showed the feature selection process for different features sets. Without dose features, other features can get prediction power around 0.55. The value of the training and testing AUC are presented in manuscript.


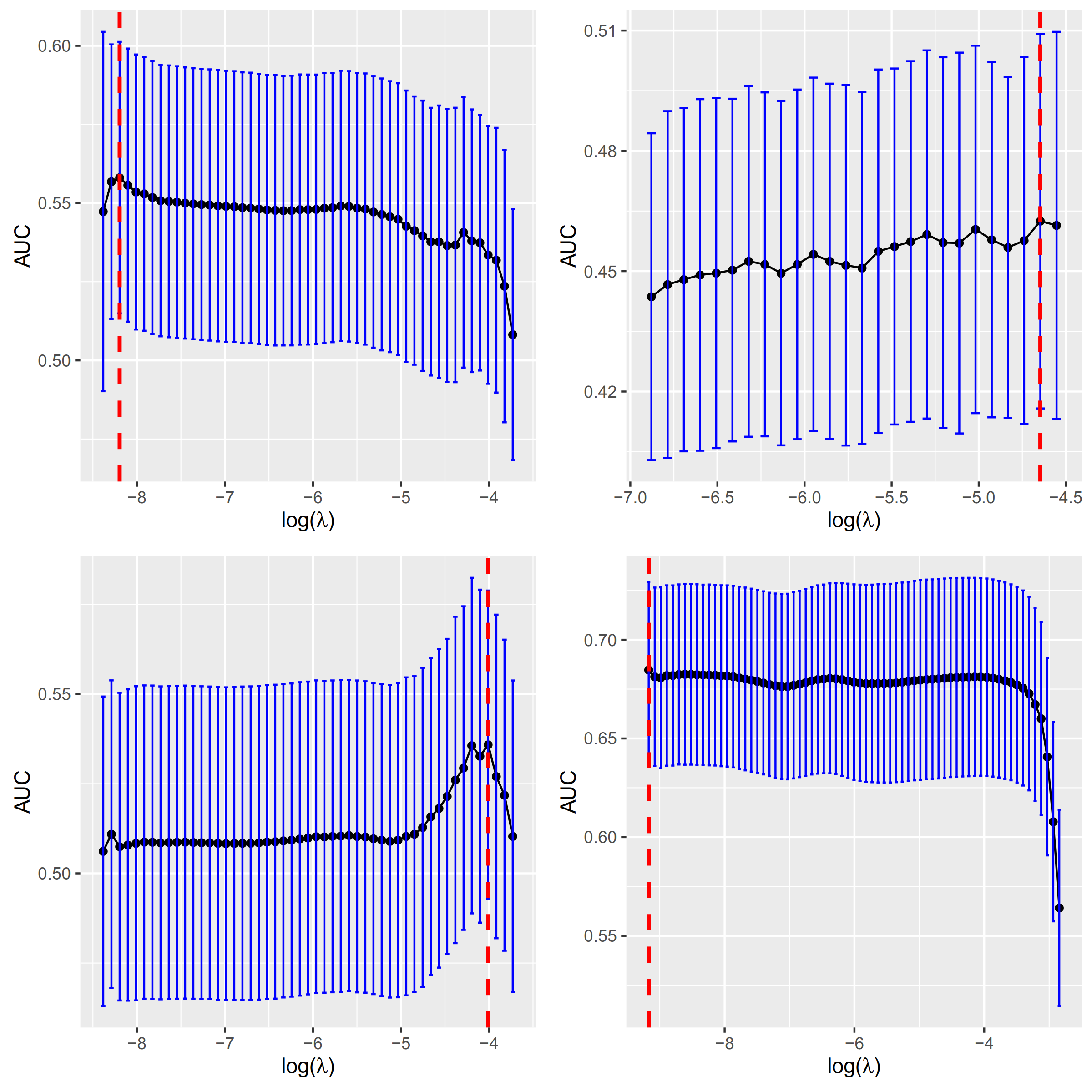


Figure s5. Feature selection using the least absolute shrinkage and selection operator for a. diagnosis features, b. treatment features. c. diagnosis and treatment features. d. dose features
